# Is sleep position associated with glenohumeral shoulder pain and rotator cuff tendinopathy: a cross-sectional study

**DOI:** 10.1186/s12891-018-2319-9

**Published:** 2018-11-23

**Authors:** Lincoln A. Holdaway, Kurt T. Hegmann, Matthew S. Thiese, Jay Kapellusch

**Affiliations:** 1 Rocky Mountain Center for Occupational and Environmental Health, University of Utah, 391 Chipeta Way, Suite C, Salt Lake City, UT 84108 USA; 2 Department of Occupational Science and Technology, University of Wisconsin-Milwaukee, 161 W Wisconsin Ave, Suite 6000, Milwaukee, WI 53203-2602 USA

**Keywords:** Sleep position, Rotator cuff tendinopathy, Glenohumeral pain

## Abstract

**Background:**

Glenohumeral pain and rotator cuff tendinopathy (RCT) are common musculoskeletal complaints with high prevalence among working populations. The primary proposed pathophysiologic mechanisms are sub-acromial RC tendon impingement and reduced tendon blood flow. Some sleep postures may increase subacromial pressure, potentially contributing to these postulated mechanisms. This study uses a large population of workers to investigate whether there is an association between preferred sleeping position and prevalence of: (1) shoulder pain, and (2) rotator cuff tendinopathy.

**Methods:**

A cross-sectional analysis was performed on baseline data from a multicenter prospective cohort study. Participants were 761 workers who were evaluated by questionnaire using a body diagram to determine the presence of glenohumeral pain within 30 days prior to enrollment. The questionnaire also assessed primary and secondary preferred sleep position(s) using 6 labeled diagrams. All workers underwent a structured physical examination to determine whether RCT was present. For this study, the case definition of RCT was glenohumeral pain plus at least one of a positive supraspinatus test, painful arc and/or Neer’s test. Prevalence of glenohumeral pain and RCT were individually calculated for the primary and secondary sleep postures and odds ratios were calculated.

**Results:**

Age, sex, Framingham cardiovascular risk score and BMI had significant associations with glenohumeral pain. For rotator cuff tendinopathy, increasing age, Framingham risk score and Hand Activity Level (HAL) showed significant associations. The sleep position anticipated to have the highest risk of glenohumeral pain and RCT was paradoxically associated with a decreased prevalence of glenohumeral pain and also trended toward being protective for RCT. Multivariable logistic regression showed no further significant associations.

**Conclusion:**

This cross-sectional study unexpectedly found a reduced association between one sleep posture and glenohumeral pain. This cross-sectional study may be potentially confounded, by participants who are prone to glenohumeral pain and RCT may have learned to avoid sleeping in the predisposing position. Longitudinal studies are needed to further evaluate a possible association between glenohumeral pain or RCT and sleep posture as a potential risk factor.

## Background

Glenohumeral pain is the third most common musculoskeletal complaint, with Rotator Cuff Tendinopathy (RCT) being the most commonly diagnosed cause of shoulder pain [1]. The prevalence of these conditions has been estimated from 4.5% among workers [2] to as high 47% in another study with a more general case definition [3]. Economic costs of RC tendinopathy are high due to loss of productivity, absenteeism, and direct healthcare costs [1]. Shoulder injuries represented 7.1% of Washington state worker’s compensation claims and had the highest median costs per claim of all work-related musculoskeletal disorders at $28,228 [4].

Risk factors for RCT appear to include age, sex, obesity, Framingham cardiovascular disease score, smoking, and psychosocial factors [5–8]. Suggested occupational risk factors include force, posture, repetition and vibration [9], but the overall quality of ergonomic-epidemiological studies tends to be poor [10]. There are two main pathophysiologic disease mechanisms proposed: sub-acromial impingement of the RC tendons [11], and reduced blood flow with hypovascularity of the RC tendons [12–14].

Sleep position has been theorized as a possible risk factor for the development of RCT and its potential impacts may share a mechanism with the two theoretical pathophysiological mechanisms. Catheter measurements of subacromial pressures during four common sleep positions have shown lower subacromial pressures in a supine sleeping position, while prone or side sleeping positions with the arms overhead have higher pressures [15]. Zenian, noting that many first experience shoulder pain upon awakening, also found a relationship between laterality of shoulder pain and laterality of sleep position [16].

This study utilizes a large population of workers with diverse job physical demands to investigate whether there is an association between preferred sleeping position and the prevalence of: (1) shoulder pain, and (2) rotator cuff tendinopathy. To our knowledge, no study has been performed on a large, diverse occupational cohort that analyzes a possible relationship between sleep position and shoulder pain or RCT.

## Methods

This study is a cross-sectional analysis of baseline date collected from a multicenter prospective cohort study of upper extremity musculoskeletal disorders—the WISTAH study. Detailed study methods are published by Garg et al. [17], thus brief methods follow.

### Participants

Workers were recruited from 15 employers with 17 distinct production facilities located in Wisconsin, Utah, and Illinois, USA. The workers were not incentivized to participate, but were paid normal wages during the study. All participants signed informed consent documents. The study’s enrollment goal was to recruit approximately one-third of workers into low, medium, and high job physical demand groups.

### Data collection

**Fig. 1:**
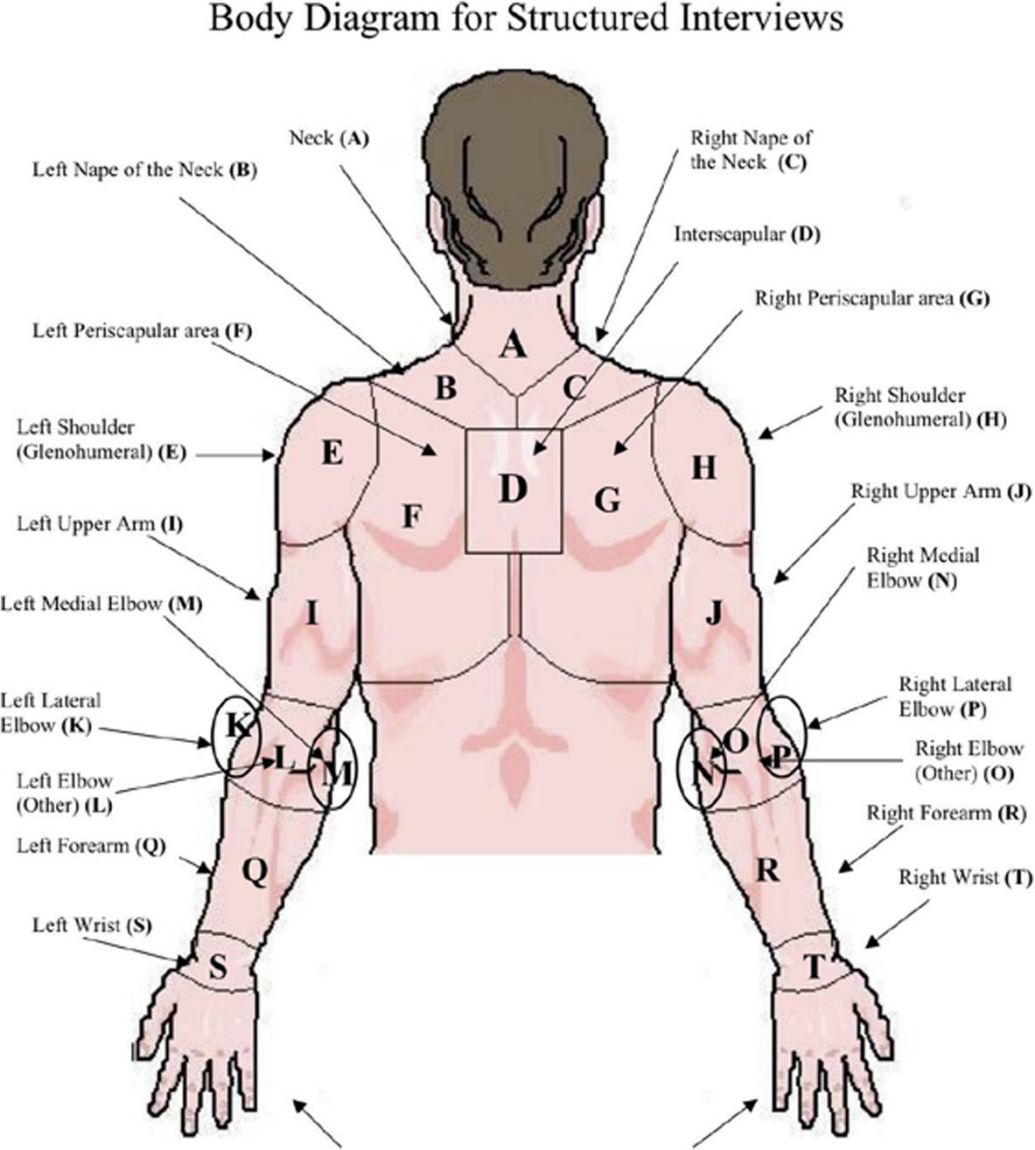
Body Diagram used to Locate Symptoms during the Structured Interview. Areas E and H are glenohumeral pain

Two independent and blinded teams collected outcomes and exposure data. The Health Assessment Outcomes Team administered computerized questionnaires, structured interviews, vital signs measurements, and physical examinations. The computerized questionnaire included basic demographics (e.g., age, gender, maximum body weight), habits (e.g. smoking), and medical history. The questionnaire was also used to ascertain sleep position while referencing Fig. 1 with the question: “Choose and rank up to two (2) of your most common sleeping postures.”

**Fig. 2:**
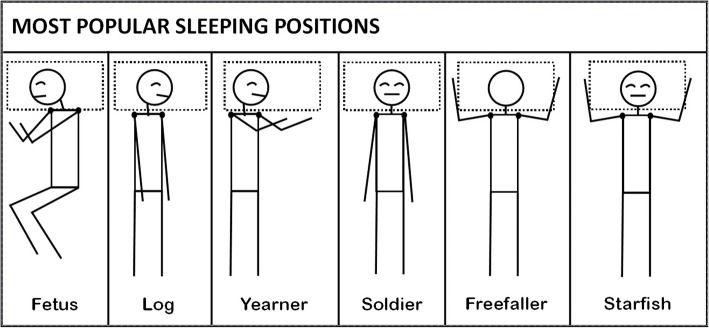
Preferred Sleep Position Diagrams Used by Workers to Report Sleep Positions* *(computer survey identified positions alphabetically rather than by named descriptors, e.g., “C” or “D”)

The computerized structured interview included a symptoms survey. A body diagram was used to help workers locate their symptoms. The body diagram differentiated shoulder pain between right and left shoulder, and separated the shoulder into the following areas: interscapular, nape of the neck, periscapular, glenohumeral shoulder, and upper arm (see Fig. 2). This structured interview ascertained symptoms within the past month. Histories of prior diagnosed disorders were also collected including: rotator cuff tear, rotator cuff tendinitis, shoulder dislocation, and surgical history. Next, vital signs were measured including height and weight to calculate body mass index (BMI).

Two independent standardized physical examinations were conducted by a team of examiners that included hand therapists, occupational medicine residents, and board-certified occupational medicine physicians. All examiners were standardized by reviewing a videotaped examination and were subsequently trained on examination techniques in training sessions to ensure examination reproducibility.

Assessments began with a structured interview followed promptly by an initial physical examination. Both of these were performed either by a hand therapist or occupational medicine resident. The first physical examination included all examination maneuvers, regardless of the presence or absence of symptoms. A second physical examination was performed by a board-certified occupational medicine physician so as to confirm positive and evaluate pertinent negative findings of the initial examination. Neither examiner was blinded to symptoms, and the second examiner was not blinded to the results of the initial physical examination.

Normal or abnormal results were recorded for each examination test separately on each shoulder: painful arc test, impingement sign (Neer test), supraspinatus test (empty can test). Only pain elicited in the glenohumeral joint was considered positive for these diagnostic maneuvers. Thus, elicitation of only upper trapezius pain was recorded as “negative.”

The Job Physical Exposure Assessment Team measured each worker’s baseline job physical demands. Data were collected via interview, observation and videotape analyses. Using this information, the Strain Index (SI) [18] and the Threshold Limit Value for Hand Activity Level (TLV-HAL), a composite of force and repetition, were calculated for each worker. The SI was also calculated and is a composite measure of the job’s requirements of force, repetition rate, duration of exertion, hand/wrist posture, speed of work and hours per day. While the SI and TLV-HAL were designed for distal upper extremity physical exposure assessment, the SI has been shown to be associated with shoulder tendinitis [19] suggesting that hand/wrist and shoulder physical exposures are correlated.

### Case definition

The case definition for RCT was: (1) self-reported glenohumeral pain within the past month, and (2) at least one positive shoulder examination finding (i.e., painful arc, supraspinatus, and/or Neer’s tests). The case definition for glenohumeral pain was any self-reported glenohumeral pain irrespective of physical exam findings.

## Statistical analyses

Statistical analyses for this report were: 1) prevalence of glenohumeral pain and rotator cuff tendinopathy, 2) prevalences of glenohumeral pain and RCT each stratified into each of the sleep positions, 3) odds ratio for associations between sleep position and RCT and glenohumeral pain. Significant associations are reported based on two-sided statistical significance testing.

As sleeping with a neutral arm position (see Fig. 1d.) is theorized to have the lowest glenohumeral joint pressures, data for that position were used to compare with other individual and aggregated sleep positions using chi-square analyses. Overhead arm positions, Fig. 2 positions ‘freefaller’ and ‘starfish’, are compared with the supine position (soldier) as overhead positions best fit the impingement model.

To evaluate the combined effect of primary and secondary sleep positions, a predictive model was created. While blinded to results, each sleep position was a priori assigned a rank number based on its suspected impact on glenohumeral pain and RCT. The value of each ranked position score was combined by adding two thirds the value of the person’s primary sleep position to one third the value of the secondary sleep position. Workers who did not identify a secondary sleep position were given the full value of their primary sleep position. The results were divided into quartiles and tested for association with chi square testing.

Multivariate logistic regression was used to assess for covariates. The following variables were a priori evaluated as potential confounders: age, sex, and BMI. The SI and HAL are evaluated as potential effect modifiers, as higher intensity jobs may theoretically predispose to shoulder tendinopathy. As one of the theories for RCT is reduced blood flow to the tendons, cardiovascular disease risk [6] and smoking [5] were also evaluated as possible effect modifiers.

## Results

There were 761 workers with complete data included in these analyses. The average age was 41.8 (±11.1) years (see Table 1). Most workers (68.9%) were female. The average BMI of all workers was 29.7 (±6.8) kg/m^2^ and the average Framingham risk score was 6.13 (±5.1). Prevalence of self-reported glenohumeral pain in one or both shoulders in the 30 days prior to enrollment was 36.4% (*n* = 277 workers). Regarding the case definition for RCT, there were 18.0% (*n* = 137) who had both glenohumeral pain and at least one positive physical examination maneuver in one or both shoulders.

**Table 1:** Demographics of the included workers

Demographics	Mean ± SD, or N (%)
Age	41.8 ±11.1 years
Sex	
Male (237)	237 (31.1)
Female (524)	524 (68.9)
Glenohumeral Pain (in either shoulder reported in the previous 30 days)	277 (36.4)
Rotator Cuff Tendinopathy (in either shoulder)	137 (18.0)
Body Mass Index (BMI) Kg/m^2^	29.7±6.8
Underweight > 18.5	3 (0.4)
Normal Weight 18.5–25	202 (26.5)
Overweight 25–30	241 (31.7)
Obese > 30	315 (41.4)
Tobacco Use	
Current use	211 (27.7)
Previous use	185 (24.3)
Never	362 (47.6)
Missing	3 (0.4)
Framingham Risk Score	6.13±5.1
Hand Activity Level (HAL) Left	0.64 ±0.6
Hand Activity Level (HAL) Right	0.66±0.6
Strain Index (SI), Left	7.7±9.9
Strain Index (SI), Right	9.3±10.8

Workers with glenohumeral pain were significantly older than those without (44.4 (±10.3) years compared with 40.3 (± 11.3) years, see Table 2). Similarly, workers with glenohumeral pain had higher BMIs (30.6 (±7.2) versus 29.2 (±6.6) kg/m^2^), and higher Framingham risk scores (7.39 (± 5.3) compared to 5.43 (±4.8)). There were no statistical differences in tobacco use or occupational physical exposure as measured by the SI and TLV-HAL (Table 2).

**Table 2:** Comparison of workers with and without glenohumeral shoulder pain

Demographics	Glenohumeral Shoulder Pain	No Glenohumeral Shoulder Pain	Statistical Test^a^
	*N* = 277 ± SD, (%)	*N* = 484 ± SD, (%)	
Age	44.41 ± 10.3	40.3 ± 11.3	*p* < 0.01
Sex			
Male (237)	66 (23.8)	171 (35.33)	*P* < 0.01
Female (524)	211 (76.2)	313 (64.67)	
Body Mass Index (BMI) Kg/m^2^	30.6 ± 7.2	29.2 ± 6.6	*P* < 0.01
Underweight > 18.5	0	3 (0.62)	*p* = 0.02
Normal Weight 18.5–25	57 (20.6)	145 (30.0)	
Overweight 25–30	92 (33.2)	149 (30.8)	
Obese > 30	128 (46.2)	187 (38.6)	
Tobacco Use			
Current use	82 (29.6)	129 (26.7)	*p* = 0.43
Previous use	58 (20.9)	127 (26.2)	
Never	136 (49.1)	226 (46.7)	
Missing	1 (0.4)	2 (0.4)	
Framingham Risk Score	7.39 ± 5.3	5.43 ± 4.8	*p* < 0.01
Hand Activity Level (HAL) Left	0.66 ± 0.6	0.64 ± 0.6	*p* = 0.66
Hand Activity Level (HAL) Right	0.66 ± 0.6	0.66 ± 0.6	*p* = 0.88
Strain Index, Left	7.89 ± 11.2	7.59 ± 9.1	*p* = 0.70
Strain Index, Right	8.88 ± 9.0	9.47 ± 11.8	*p* = 0.48

^a^T-test for continuous data, Chi square for categorical data, Wilcoxon rank sum for non-parametric data (sex and BMI)

Workers with RCT were statistically older than those without (45.0 (±11.0) versus 41.1 (±11.0) years, Table 3). Similarly, workers with RCT had significantly higher mean Framingham scores (7.89 (±5.5) versus 5.75 (±4.9)) and had relatively higher BMI, but that difference was not statistically significant (*p* = 0.081). Contrary to glenohumeral pain, workers with RCT had statistically higher average TLV-HAL scores. There were no statistically significant differences between the two groups in regards to the SI scores.

**Table 3:** Comparison of workers with and without Rotator Cuff Tendinopathy (RCT)

Demographics	Rotator Cuff Tendinopathy	No Rotator Cuff Tendinopathy	Statistical Test^a^
	*N* = 137 ± SD, (%)	*N* = 624 ± SD, (%)	
Age	45.0 ±11.0	41.1±11.0	*p* < 0.01
Sex			
Male (237)	35 (25.6)	202 (32.4)	*p* = 0.12
Female (524)	102 (74.5)	422 (67.6)	
Body Mass Index (BMI) Kg/m^2^	29.9±6.2	29.7±7.0	*p* = 0.33
Underweight > 18.5	0	3 (0.5)	*p* = 0.08
Normal Weight 18.5–25	25 (18.3)	177 (28.4)	
Overweight 25–30	49 (35.8)	192 (30.8)	
Obese > 30	63 (46.0)	252 (40.4)	
Tobacco Use			
Current use	37 (27.0)	174 (27.9)	*p* = 0.82
Previous use	35 (25.6)	150 (24.0)	
Never	64 (46.7)	298 (47.8)	
Missing	1 (0.7)	2 (0.3)	
Framingham Risk Score	7.89±5.5	5.75±4.9	*p* < 0.01
Hand Activity Level (HAL) Left	0.80±0.8	0.61±0.5	*P* < 0.01
Hand Activity Level (HAL) Right	0.80±0.8	0.63±0.5	*P* < 0.01
Strain Index (SI), Left	7.31±6.9	7.79 ± 10.5	*p* = 0.69
Strain Index (SI), Right	9.23±9.4	9.25±11.1	*p* = 0.51

^a^T-test for continuous data, Chi square for categorical data, Wilcoxon rank sum for non-parametric data (sex and BMI)

There was an association between primary sleep position of ‘freefaller’ and glenohumeral pain; however, no other significant associations were found (see Table 4). No statistically significant associations were found between primary sleep position and RCT. Similarly, no secondary sleep positions demonstrated significant relationships with glenohumeral pain or RCT (data not shown). In evaluating laterality, if a worker had left shoulder pain, they were 2.06 (95% CI 1.07–3.96) times more likely to sleep on the right side; if they had right shoulder pain, they were 0.64 (0.35–1.20) times more likely to sleep on the left side. Laterality of sleep posture was not significantly affected by the TLV for HAL (*p* = 0.719) or SI (*p* = 0.172).

**Table 4:** Prevalences and univariate associations of pain and rotator cuff tendinopathy with sleep position

Demographics	Glenohumeral Shoulder Pain 277, (%)	No Glenohumeral Shoulder Pain 484, (%)	OR (CI)	Rotator Cuff Tendinopathy 137, (%)	No Rotator Cuff Tendinopathy 624, (%)	OR, (CI)
Sleep Position #1						
Fetus	163 (58.8)	268 (55.4)	~	81 (59.1)	350 (56.1)	~
Freefaller	26 (9.39)	73 (15.1)	0.59 (0.36–0.95)	15 (11.0)	84 (13.5)	0.77 (0.42–1.41)
Log	8 (2.89)	25 (5.1)	0.53 (0.23–1.19)	4 (2.9)	29 (4.7)	0.60 (0.20–1.74)
Soldier	30 (10.83)	39 (8.1)	1.26 (0.76–2.11)	12 (8.8)	57 (9.1)	0.91 (0.47–1.77)
Starfish	18 (6.50)	41 (8.5)	0.72 (0.40–1.30)	9 (6.6)	50 (8.0)	0.78 (0.37–1.65)
Yearner	32 (11.55)	38 (7.9)	1.38 (0.83–2.30)	16 (11.7)	54 (8.7)	1.28 (0.70–2.35)

Comparing ‘soldier’, the hypothesized lowest risk sleep position, to all other sleep positions demonstrated no statistically protective association (OR = 1.38, 95% CI 0.84–2.29, and OR = 0.95, 95% CI: 0.50–1.83 for glenohumeral pain and RCT, respectively). Moreover, the soldier sleep position trended towards higher prevalence among those with glenohumeral pain although not at a statistically significant level. Comparing ‘freefaller’ and ‘starfish’, the hypothesized highest risk sleep position, with ‘soldier’ demonstrates significantly higher prevalance of glenohumeral pain in the hypothesized lowest risk group (*p* = 0.02, OR = 0.50, 95% CI 0.28–0.90). There was no association for this comparison in the RCT comparison (*p* = 0.68, OR = 0.85, 95% CI 0.40–1.82).

The predictive model assigning numerical values to the various sleep positions and weighting primary and secondary sleep positions to ordinally rank the hypothesized sleep risk divided into quartiles and statistically evaluated with chi-square analysis showed no increase in RCT or glenohumeral pain for the higher predicted values (see Table 5). In the glenohumeral pain comparison, the second group, which primarily represented the fetal sleep position, approached statistical significance.

**Table 5:** Chi-squared analysis of primary and secondary predicted sleep position risk scores grouped by quartile for glenohumeral shoulder pain and Rotator Cuff Tendinopathy*

Predictive model	ALL	Glenohumeral Shoulder Pain	No Glenohumeral Shoulder Pain		Rotator Cuff Tendinopathy	No Rotator Cuff Tendinopathy	
		*N* = 277	*N* = 484		*N* = 137	*N* = 624	
Group				chi = 0.07			chi = 0.24
1 (0–3.33)	123 (16.2)	44 (15.9)	79 (16.3)		20 (14.6)	103 (16.5)	
2 (4)	265 (34.8)	111 (40.1)	154 (31.8)		58 (42.3)	207 (33.2)	
3 (4.67–6)	207 (27.2)	73 (26.4)	134 (27.7)		33 (24.1)	174 (27.9)	
4 (6.67–10)	166 (21.8)	49 (17.7)	117 (24.2)		26 (19.0)	140 (22.4)	

*Risk scores for glenohumeral pain and RCT were created by assigning points to each sleep position in rank with suspected risk for increased subacromial pressures and impingement. Zero was assigned to the hypothesized lowest risk sleep position, soldier, and 10 was assigned to the hypothesized highest risk sleep position, freefaller. The sleep position log, fetus, yearner and starfish were assigned 2,4,6,8, respectively. The overall risk score was calculated by adding two-thirds of the primary sleep positions risk score to one-third the secondary sleep position’s risk score

## Discussion

We hypothesized that overhead sleep positions and sleeping on the side increase risk of glenohumeral shoulder pain and RCT. However, no association with higher prevalence of these shoulder conditions was found in this study. On the contrary, the hypothesized highest risk sleep positions (freefaller and starfish) trended towards a protective association.

Prior cross-sectional studies reported that shoulder pain was associated with laterality of sleeping [20]. So far as we are aware, this is the first study to evaluate associations with specific sleep positions. As such, it adds to the body of evidence evaluating sleep position as possible risk and protective factors for glenohumeral shoulder pain.

Similar to previous studies, this study demonstrated associations between age, BMI, and cardiovascular risk factors with glenohumeral pain and RCT [6]. As anatomic and epidemiologic evidence of vascular impairment of the supraspinatus tendon mounts, this highlights the necessity of adjusting for cardiovascular disease risk factors in assessing risk of other exposures.

It is possible that the cross-sectional nature of this study missed a relationship as glenohumeral shoulder pain and RCT may directly affect sleep positions and confound the study. It is also possible that other factors such as lack of distraction at night and daytime activity may have influenced the results. Prospective studies may be required to further evaluate possible association(s) between sleep position and either glenohumeral pain and/or RCT. Conversely, sleep position may not play a meaningful role in the pathophysiology of glenohumeral pain and RCT. This requires further investigation, particularly as a significant contribution, or alternatively, a significant protective effect, has potential clinical utility that needs to be understood and reported. For example, patient presentations prominently include sleep disturbances and guidance on that exposure-response relationship would be instructive.

### Strengths/weaknesses

There are several study strengths, including the large sample that has diverse occupational exposures. The computerized assessments of demographic variables, computerized capture of sleep positions, computerized structured interviews, and standardized physical examinations producing complete datasets were particular study strengths. The primary limitation is this study’s cross-sectional design, with the possibility that pain induced alterations in sleep position confounded the association(s) between sleep position and glenohumeral pain and/or RCT. Thus, there may be lower prevalence of glenohumeral shoulder pain in the predicted highest risk sleep position (freefaller) not because it is protective, but because individuals with these painful conditions may not tolerate that sleep position. Some potential variables (pillows, mattress firmness, sleeping alone, sleep quality) were not collected and could have had some impact. Subjective rather than objective time spent in sleep positions could have impacted findings. We also did not query whether sleep posture had changed because of the pain. Longitudinal analyses to fully understand these relationships is required.

## Conclusion

This cross-sectional study found no correlation between sleep position and glenohumeral pain or RCT. Rather, the a priori highest risk sleep position trended towards a protective effect. The absence of a positive association with the highest risk sleep position is surprising as previous studies have found increased sub-acromial pressures with overhead arm positions and certain sleeping positions that might be anticipated to lead to glenohumeral pain and RCT. The cross-sectional nature of this study may have missed a true relationship if these workers altered sleep position as a result of glenohumeral shoulder pain and RCT, thus confounding the study. A prospective cohort study is needed to further evaluate sleep position as a possible risk factor for glenohumeral pain and rotator cuff tendinopathy.

## Abbreviations

BMI: Body Mass Index
CI: Confidence Interval
HAL: Hand Activity Level
RC: Rotator Cuff
RCT: Rotator Cuff Tendinopathy
SI: Strain Index
TLV-HAL: Threshold Limit Value for Hand Activity Level
